# Clinical Application of Foldable Capsular Vitreous Bodies in the Treatment of Severe Ocular Trauma and Silicone Oil Dependent Eyes

**DOI:** 10.1155/2022/3608162

**Published:** 2022-10-28

**Authors:** Hao Jiang, Chao Xue, Yanlin Gao, Ying Chen, Yan Wang

**Affiliations:** ^1^Tianjin Eye Hospital, Tianjin Key Laboratory of Ophthalmology and Visual Science, Tianjin Eye Institute, Tianjin, China; ^2^Nankai University Affiliated Eye Hospital, Nankai University, Tianjin, China; ^3^Clinical College of Ophthalmology, Tianjin Medical University, Tianjin, China

## Abstract

**Purpose:**

This study aimed to assess the application of a foldable capsular vitreous body (FCVB) in the treatment of severe ocular trauma and silicone oil (SO) dependent eyes.

**Methods:**

A retrospective analysis was performed on the clinical application of FCVB in the treatment of severe ocular trauma and SO dependent eyes. The results of best-corrected visual acuity and intraocular pressure (IOP) evaluation, B-scan ultrasonography or color Doppler ultrasonography, ultrasound biomicroscopy, and anterior segment photography were recorded during follow-up. A paired *t*-test was used to compare the difference in IOP before and after FCVB implantation.

**Results:**

Seven eyes of seven patients were included in the 6-month follow-up. In all cases, B-scan ultrasonography and ultrasound biomicroscopy showed that FCVB adapted closely to the globe wall and ciliary body, thus supporting the retina. Visual acuity did not improve, except in one case from LP to HM. The mean ± SD IOP was 8.5 ± 1.90 mm·Hg prior to FCVB implantation and 10.43 ± 0.98 mm·Hg after implantation, with no significant difference between these measurements (*P*=0.095). Five of the seven patients developed differing degrees of corneal opacity and keratopathy.

**Conclusions:**

FCVB implantation may be a safe and effective method for the treatment of severe ocular trauma and SO dependent eyes. However, FCVB cannot prevent the phthisis of the traumatic eyes. In addition, corneal opacity and keratopathy are potentially serious complications after surgery. Appropriate case selection and proper surgical timing are required for further investigation.

## 1. Introduction

Since the 1970s [1], pars plana vitrectomy has been used to treat a number of ocular diseases that were previously regarded as immedicable, such as proliferative diabetic retinopathy, proliferative vitreoretinopathy, traumatic proliferative vitreoretinopathy, and endophthalmitis [2–6]. In recent years, the technique of vitrectomy has developed rapidly, and indications are increasing.

As the vitreous body is unable to regenerate, an adequate substitute is required to ensure homeostasis of the eye after removing the native vitreous during vitreoretinal surgery [7]. Several artificial vitreous substitutes, such as inert gas, silicone oil (SO), and heavy SO, have been used clinically [8–11]. However, these vitreous substitutes have several limitations, including short residence time, the elevation of intraocular pressure (IOP), cataracts, emulsification, keratopathy, and secondary glaucoma [12–14].

Recently, a new type of foldable capsular vitreous body (FCVB) has been developed for clinical application. It is flexible, effective, and safe as a vitreous substitute and may avoid direct contact with intraocular tissue and reduce complications associated with vitreous substitutes. Therefore, it may be an ideal substitute for the vitreous body in eyes with severe retinal detachment [15–19]. Nevertheless, for all new clinical products, further studies are necessary. The purpose of this study was to summarize our experience with the application of FCVB in the treatment of severe ocular trauma or SO dependent eyes.

## 2. Methods

As this was the first cohort of patients undergoing this surgery, we established strict inclusion criteria. All patients had a history of severe ocular trauma, had undergone several operations, had an IOP which could not be maintained with SO tamponade, and satisfied the requirements of FCVB implantation. The exclusion criteria were contraindications approved by the China Food and Drug Administration. The surgical technique has been described previously [19, 20].

The results of best-corrected visual acuity and IOP evaluation, B-scan ultrasonography or color Doppler ultrasonography, ultrasound biomicroscopy, and anterior segment and fundus photography were recorded in this study. Terms used in the description of ocular injuries conform to the recommendations of the American Academy of Ophthalmology, the United States Eye Injury Registry, and the International Society of Ocular Trauma [21].

A paired *t*-test was used to compare the difference in IOP before and after FCVB implantation.

This study was approved by the Ethics Committee of Tianjin Eye Hospital, Tianjin Medical University. Informed consent was obtained from the patients and their families before any examination or treatment was performed.

## 3. Results

Seven eyes of seven patients were included in this study. The characteristics of the seven patients are summarized in Table 1. The participants included six men and one woman, with a mean age of 38.57 ± 11.70 years (range, 17 to 54 years).

**Figure 1:**
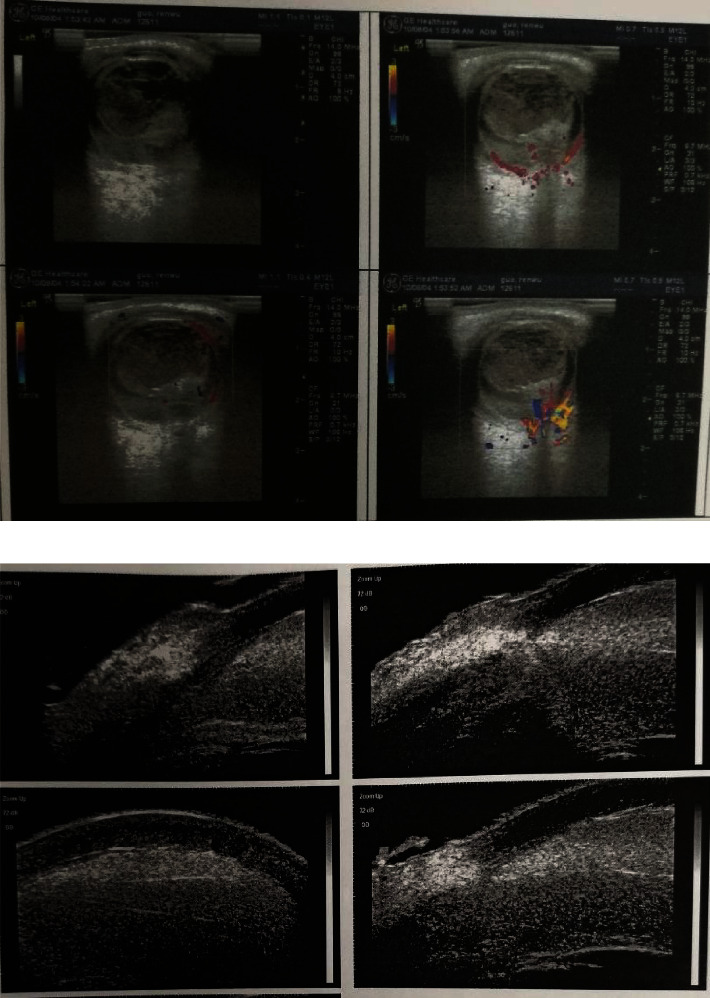
(a): Color Doppler ultrasonography of a patient exhibiting vitreous fibroplasia and choroidal and retinal detachment 3 days after repair of the globe. (b): Ultrasound biomicroscopy of a patient showing fibroplasia in the anterior chamber.

All patients had a history of severe ocular trauma and had undergone several surgeries prior to FCVB implantation (Table 1). Figures 1(a) and 1(b) show the severe eye injuries in these cases.

In this study, the last follow-up was at 6 months after FCVB implantation. Among these seven eyes, two eyes underwent four surgeries, four eyes underwent three surgeries, and one eye underwent two surgeries. The time between SO injection and FCVB implantation was 5.29 ± 3.33 months (range, 3–13 months). In the first case, the FCVB was refilled with SO due to low IOP because of postoperative oil leakage in the first month after surgery; the IOP remained stable after refilling.

**Figure 2:**
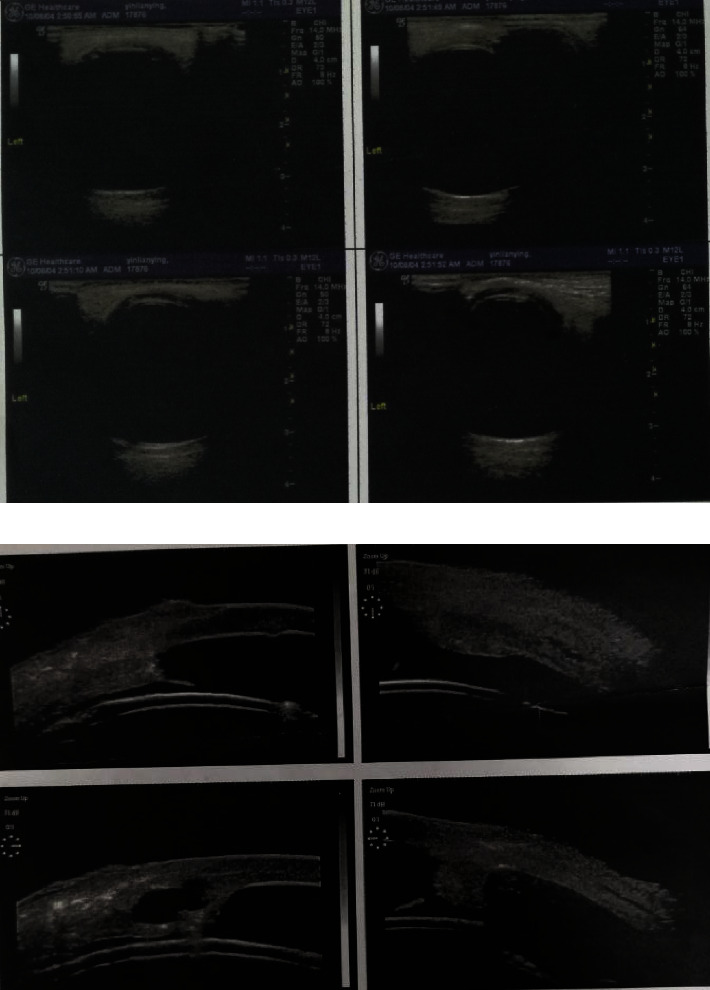
(a): Color Doppler ultrasonography of a patient 6 months after FCVB implantation. (b): Ultrasound biomicroscopy of a patient 6 months after FCVB implantation.

In all cases, B-scan ultrasonography and ultrasound biomicroscopy showed that the FCVB adapted closely to the globe wall and ciliary body and supported the retina well. As shown in Figure 2(a), a layer of the mechanical membrane could be seen on the anterior and posterior surfaces, the FCVB position was positive, and the FCVB was well distributed within the vitreous cavity, which adequately supported the retina.  Figure 2(b) shows images six months after FCVB implantation where the FCVB capsule membrane reflex can be seen and the FCVB contacting, but not oppressing, the ciliary body is visible.

Visual acuity did not improve, except in one case from LP to HM. Changes in IOP after FCVB implantation are shown in Table 1. The mean ± SD IOP was 8.5 ± 1.90 mm·Hg prior to FCVB implantation and 10.43 ± 0.98 mm·Hg after implantation. Although IOP was higher postoperatively, there was no significant difference (*P*=0.095).

**Figure 3:**
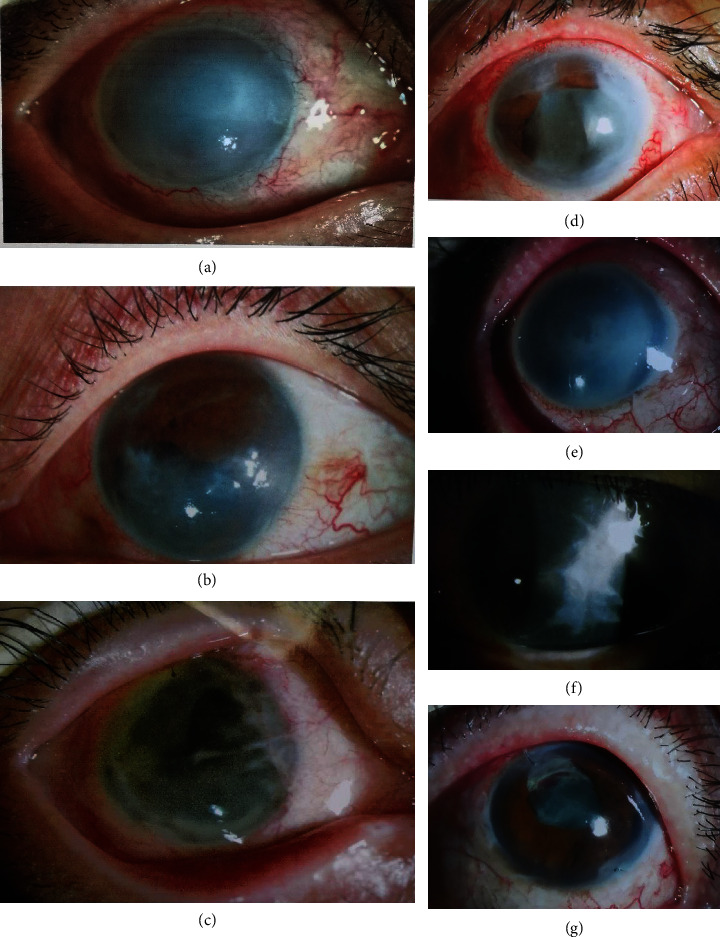
Anterior segment photography of the 7 cases at 6 months after FCVB implantation.

In addition, it is worth noting that five of the seven patients developed severe corneal opacity or keratopathy midway during postoperative follow-up (Figure 3). In the two cases (cases 2 and 7) with relatively good corneal transparency, the time between SO injection and FCVB implantation was no more than three months.

## 4. Discussion

Proliferative vitreoretinopathy is a highly probable consequence of severe ocular trauma, which usually requires pars plana vitrectomy to prevent disease progression. As the vitreous body is unable to regenerate, an adequate substitute is required to ensure homeostasis of the eye after removing the native vitreous during vitreoretinal surgery [7]. Therefore, the introduction of an optimal vitreous substitute in the course of a vitrectomy is essential. SO is the most widely used intraocular tamponade in clinical practice. However, it has many drawbacks and limitations, such as elevated IOP, oil emulsification, secondary glaucoma, and keratopathy [12, 22, 23].

FCVB is a new product that has refined the way in which SO works in the inner cavity of the eyeball [19]. It has been reported that it can prevent the displacement and emulsification of SO [15] and effectively reduce postsurgical complications [17]. FCVB filled with SO has been shown to be effective and safe in humans' eyes [17]. However, we have identified some issues with FCVB in clinical practice. The purpose of this study was to summarize our experience with the application of FCVB.

As FCVB is a relatively new product and was not used at our hospital previously, we were very careful in selecting appropriate patients. All cases in this study had a history of severe ocular trauma and underwent several operations, and IOP could not be maintained with SO tamponade. Judging from our clinical experience, we initially selected only cases with no light perception or visual acuity and cases of silicone oil-dependent eyes. All the cases in this study had severe ocular injuries, and the prognoses were very poor.

In this study, with the exception of the shallow anterior chamber, no other structural abnormalities were found after FCVB implantation. B-scan ultrasonography revealed that the FCVB was in good contact with the retina and had good retina-supporting function. Furthermore, no retinal detachment was observed during the follow-up. Ultrasound biomicroscopy showed that the FCVB smoothly contacted the ciliary body with no crushing action.

Visual acuity did not improve after FCVB implantation in this study, which is consistent with the results of previous studies [15, 18, 19].

No significant differences were found in IOP after FCVB implantation, although it was higher postoperatively. In clinical practice, we have observed that postoperative IOP is mainly determined by the function of the ciliary body rather than by the SO injected into the FCVB. Moreover, we do not suggest an excessive injection of SO; the amount of SO injected into the FCVB is usually less than that injected into the vitreous body directly, and an IOP of 15 mm·Hg may be appropriate. Patients with an iris must have space in the posterior chamber to avoid a shallow anterior chamber.

We did not encounter severe surgical complications during the follow-up period. A cataract was not found as the lens was lost in the primary injury or lensectomy was performed during the par plana vitrectomy surgery in this study. Other complications such as uveitis, vitreous hemorrhage, endophthalmitis, retinal detachment, and SO emulsification were not observed during the observation period. Leakage of SO was found in one case, but after refilling with SO, the condition of the eye was stable.

However, serious complications such as corneal opacity and keratopathy were observed in this study. The reason for these may be as follows: first, all eyes in this study had severe injuries and underwent several operations. Thus, the structure of the ocular surface may have changed, leading to impaired blood supply and nutrition of the cornea [24]. Second, in this study, the time between SO injection and FCVB implantation was 5.29 ± 3.33 months. This may increase the toxic effect of SO on the corneal endothelium and ciliary body [25, 26]. Previous studies [27–29] have reported corneal perforation secondary to SO keratopathy due to poor corneal nutrition. Risk factors include a longer duration of oil in the eye, aphakia, SO in the anterior chamber, and extensive and multiple surgeries, all of which were present in our cases. Third, the function of the ciliary body may be impaired by multiple intraocular surgical procedures, inflammation, ciliary body shock, and destruction. Impaired ciliary body function can lead to persistent chronic hypotony and difficulty in the formation of a normal anterior chamber, carrying the risk of corneal opacification [30]. Therefore, it may be inferred that patients with poor ciliary body function may not be suitable for FCVB implantation.

From our cases, it is suggested that SO dependent eyes should not choose FCVB implantation for an extended amount of time between FCVB implantation and the first silicone oil injection to prevent cornea damage. However, if FCVB implantation is performed too early, the postoperative inflammatory response may be severe, and the function of the ciliary body cannot be accurately predicted. In this study, in cases with relatively good corneal transparency, the duration between the first SO injection and FCVB implantation was no more than 3 months. Therefore, we hypothesized that 3 months may be an appropriate time to implant the FCVB after filling SO for SO dependent eyes. However, this is our experience with a small sample size and requires further research.

There are a few limitations to the present study. The sample size was small, and case selection was relatively tight. In addition, a longer follow-up time is required. However, the results remain viable as they provide valuable information for further improvement of surgical outcomes.

In summary, FCVB implantation may be a safe and effective method for the treatment of severe ocular trauma or SO dependent eyes. However, FCVB cannot prevent the phthisis of the traumatic eyes. In addition, corneal opacity and keratopathy may be common postoperative complications of FCVBs for eyes with poor ciliary body function and hypotony. Appropriate case selection and proper surgical timing may prevent these side effects. Further studies with larger sample sizes and longer follow-up periods are required to evaluate the clinical efficacy of FCVB implantation.

## Figures and Tables

**Table 1:** Characteristics of 7 eyes.

No.	Age	Sex	Type of injury	Preope Va	Preope IOP (mm·Hg)	Surgeries/other therapies performed	Final BCVA	Final IOP (mm·Hg)
1	29	M	IOFB	NLP	12	1^st^: Ocular repairing 2^nd^: vitrectomy + phaco + SO injection 3^rd^: SO removal + FCVB + SO injection	NLP	10
2	17	M	Rupture	NLP	9	1^st^: Ocular repairing 2^nd^: vitrectomy + SO injection 3^rd^: SO removal + FCVB + SO injection	NLP	11
3	37	M	Penetrating	NLP	10	1^st^: Ocular repairing 2^nd^: vitrectomy + SO injection 3^rd^: SO removal + FCVB + SO injection	NLP	10
4	47	F	Contusion	LP	8	1^st^: Vitrectomy + phaco + SO injection + cyclopexy 2^nd^: SO removal + FCVB + SO injection	HM	9
5	38	M	Rupture	FC	7	1^st^: Ocular repairing 2^nd^: vitrectomy + phaco + SO injection + cyclopexy 3^rd^: FCVB + SO injection 4^th^: SO injection	FC	10
6	48	M	Rupture	LP	7	1^st^: Ocular repairing 2^nd^: Anterior vitrectomy 3^rd^: vitrectomy + phaco + SO injection + cyclopexy 4^th^: SO removal + FCVB + SO injection	LP	12
7	54	M	Rupture	HM	7	1^st^: Ocular repairing 2^nd^: vitrectomy + phaco + SO injection + cyclopexy 3^rd^: SO removal + FCVB + SO injection	HM	10

BCVA: best-corrected visual acuity; IOFB: intraocular foreign body; FC: finger counting; NLP: no light perception; LP: light perception; HM: hand motion; SO: silicone oil.

## Data Availability

The data used to support the findings of this study are available from the corresponding author upon request.
